# Lessons in participant retention in the course of a randomized controlled clinical trial

**DOI:** 10.1186/1756-0500-7-706

**Published:** 2014-10-09

**Authors:** Olubukola T Idoko, Olumuyiwa A Owolabi, Aderonke A Odutola, Olatunde Ogundare, Archibald Worwui, Yauba Saidu, Alison Smith-Sanneh, Abdoulie Tunkara, Gibbi Sey, Assan Sanyang, Philip Mendy, Martin O C Ota

**Affiliations:** Medical Research Council Unit, P. O Box 273, Banjul, The Gambia; Ekiti State University Teaching Hospital, Ado Ekiti, Nigeria; WHO Regional Office for Africa, Brazzaville, Congo

## Abstract

**Background:**

Clinical trials are increasingly being conducted as new products seek to enter the market. Deployment of such interventions is based on evidence obtained mainly from the gold standard of randomized controlled clinical trials (RCCT). A crucial factor in the ability of RCCTs to provide credible and generalisable data is sample size and retention of the required number of subjects at completion of the follow-up period. However, recruitment and retention in clinical trials are hindered by prevalent peculiar challenges in Africa that need to be circumvented. This article shares experiences from a phase II trial that recorded a high retention rate at 14 months follow-up at a new clinical trial site.

**Methods:**

Mothers bringing children less than two months of age to the health facility were given information and invited to have their child enrolled if the inclusion criteria were fulfilled. Participants were enrolled over 8 months. Trial procedures, duration and risks/benefits were painstakingly and sequentially explained to the communities, parents and relevant relatives before and during the trial period. The proportions of participants that completed or did not complete the trial were analyzed including the reasons for failure to complete all trial procedures.

**Results:**

1044 individuals received information regarding the trial of which 371 returned for screening. 300 (81%) of them who fulfilled the inclusion criteria and did not meet any exclusion criteria were enrolled and 94% of these completed the trial. Consent withdrawal was the main reason for not completing the trial largely (75%) due to the father not being involved at the point of consenting or parents no longer being comfortable with blood sampling.

**Conclusions:**

Participant retention in clinical trials remains a crucial factor in ensuring generalisability of trial data. Appropriate measures to enhance retention should include continuous community involvement in the process, adequate explanation of trial procedures and risks/benefits; and innovative tracing of participants adapted for the setting.

## Background

Clinical trials are increasingly being conducted across the world as new drugs, vaccines and other products seek to enter the market. Evidence for the safety and efficacy of interventions are mainly based on the gold standard of randomized controlled clinical trials (RCCT) to generate credible and generalisable data, and thus retaining the required number of subjects has become a key issue [1, 2]. Conducting such trials in sub-Saharan Africa is however still relatively new and encumbered with challenges.

Challenges within the region which may impact on subject retention are numerous. As the region boasts of a rich cultural heritage passed down from generation to generation, myths and traditions abound which may also have effects on new interventions and procedures. For instance, in certain parts of West Africa blood is considered sacred and children are thought to be made ill by blood sampling [3]. In parts of East Africa, hair is considered sacred and should not be knowingly removed. (Personal communication) Thus clinical trials which involve blood or hair sampling for analysis would likely be met with resistance in these areas. Such factors imply that the factors affecting subject retention may vary region to region. Other factors considered to negatively influence participant retention in clinical trials include erratic health care utilization [4], low literacy levels [5], male gender [6], older age of trial participants [7] and psychological distress [8]. Apart from these participant related factors, there are also investigator related barriers, which could be logistic or personnel factors. A common one is the failure to integrate the role as a caregiver and investigator, and failure to anticipate the required work load [9]. Other barriers include the lack of time and resources, and poor motivation of investigators. Protocol related barriers may include, lengthy trial periods, and over burdensome visit schedule requiring a significant degree of change in the participants’ routine activities [10–12]. Barriers may also include the influence of media [13, 14] or community groups. In addition, there are few established clinical trial sites in the region with limited human resources trained in running such trials. The few trial sites available are often overwhelmed leading to products in queue awaiting clinical trial and thus delayed availability of products to end users. This has led to a need to build capacity and establish new trial sites.

Studies in adults have reported attrition rates of 24% at 12 months and 44% at 24 months duration in longitudinal studies [15]. In paediatric developmental studies attrition rates of 10 to 15% are generally expected each year [16]. Such high rates of attrition of trial participants may reduce the statistical power or lead to skewed representation of data.

The Medical research Council Unit (MRC) Unit The Gambia has been conducting RCCTs, and had to establish a new site at Faji Kunda Health Centre to run another trial due to the burden on the existing site. Lessons learned from previous sites and trials were utilized in setting up this new site and interestingly the trial recorded a high participant retention rate of 94% at 14 month follow up. The culture and belief system especially related to the collection of blood samples in children, and low literacy levels were considered major factors which could likely impact participant retention at this new site. This paper highlights the operational lessons learnt in subject retention during the course of this trial with the hope that other sites may learn from these and adapt where feasible.

## Methods

**Figure 1:**
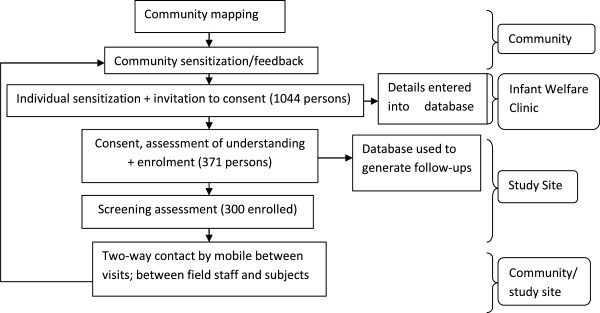
**Methods utilized.**

The trial was conducted at the Faji Kunda Health Centre that serves 7 districts within the locality in the Kombo region of The Gambia. The staff from this centre conduct outreach immunization clinics to neighboring districts. The area is peri-urban with a population of about 200,000 that are mainly subsistence farmers. This community was chosen for its close proximity to large health facilities, relatively stable non-mobile population, and fairly large estimated population to ensure that required sample size could be obtained.Following ethical approval from the Gambian Government/Medical Research Council joint Ethics Committee, the phase II trial recruited 300 infants aged 2 to 7 months at enrolment in two groups of 150 subjects each. Each of these groups was further divided into three giving a total of six sub-groups. Informed consent was obtained from the parent/guardian of each child enrolled. The trial started with the older group of children aged 5–7 months at enrollment. Following review of safety data on the first 20 participants from this group, enrollment of the younger children aged 2–3 months at enrollment commenced in parallel (Figure 1). Participants had between 6 and 9 scheduled trial visits depending on the group to which the participant was randomized, and involved the collection of 5 to 6 blood samples ranging from 1 to 6mls in volume per blood draw over the trial period. All procedures were carried out to International Conference on Harmonization- Good Clinical Practice (ICH-GCP) standards.Prior to commencement of the trial, the trial team met with the district head (Alkalo), his cabinet, women, men and youth leaders in order to get acceptance of the trial from the major stakeholders in the community. At these meetings, information regarding the trial objectives, age of potential participants, duration of participation, expected trial procedures and risks/benefits anticipated were highlighted. The community leaders then had opportunity to ask questions from the trial team. Following these discussions, the intended studies were announced at the mosques and churches within the community. Thereafter the trial team carried out extensive mapping of the trial area. This included numbering the houses, inhabitants of the houses, enumeration of number of pregnant women, number and ages of children under one year in each household, and situating the house within a map of the area. Potential participants were then identified at the Health Centre at routine infant vaccination visits and parents given information regarding the trial from trial information sheets (sensitization). A copy of the information sheet was then provided to the parent to take home to read or have read to them if illiterate, and discuss further with other decision makers in the home (Figure 1).

Diagrams and landmarks were used in documenting home addresses and where feasible, field assistants visited the homes prior to enrolment to ensure that the participant could be traced. The field staff also used this opportunity to discuss details of the trial with the other parent or other family members deemed important in the decision making process. A potential participant tracking database was then set up such that each participant sensitized for subsequent enrolment had basic details including contact mobile phone numbers entered (Figure 1). From this database potential trial participants were identified as they became eligible for enrolment through a list generated for subject follow up with an indication of the allowable time window. These potential participants’ parents were now approached and invited to a formal consent procedure at which the information contained on the participant information sheets was discussed and questions answered. Those interested in participating in the trial then had an assessment of consent information understanding using a structured open ended questionnaire. This questionnaire included an assessment of the potential participants’ parents understanding that they could withdraw from the trial at any time without adverse impact. Inability to pass this ‘test’ (failing to answer two or more questions correctly at a maximum of two attempts) implied a lack of understanding of the consent information and such a potential participant did not proceed further [17]. Those who passed this ‘test’ went on to sign the consent document and proceeded to screening for eligibility to participate in the trial. Literate impartial witnesses independent of the trial sat in on the consent process for each illiterate potential participant to attest that all required information was given, the potential participants’ parent had an opportunity to ask questions and apparently understood the discussion. This witness then filled in the portions of the form to be filled by the parent, while the parent thumb printed the form and the witness signed.

In addition each participant received an appointment card specifying the preferred date to meet with the trial team. This was backed up with manual tracking forms and lists of participants due visits which could be used should the electronic database fail. Each field assistant/nurse was equipped with a mobile phone and top-up funds were provided weekly depending on anticipated follow ups, to ensure that participants could be reached at any time as required.

Each participant was questioned regarding their availability during the follow-up period, including known short-term travel plans, and travels related to religious festivals or family events. The likelihood of moving to live with in-laws, parents or other family members was also explored as this is a common practice after child birth within the area. Where possible, visits and post vaccination follow-up visits were planned around these events and where these would interfere with follow-up or care this was explained to the parent or legal guardian to enable appropriate adjustments. Potential participants unable or unwilling to comply were excluded at the point of recruitment.

Previous utilization of health services in particular immunization clinics was assessed from the infant welfare record of each child. Potential participants who had been erratic with the utilization of immunization services were considered unlikely to comply with the trial procedures and excluded from enrollment into the trial.

Salient information from the information sheets was reinforced at each clinic visit and participants given opportunities to ask questions and confirm willingness to continue in the trial. Illustrations using specimen tubes that contained colored fluid equal to the volume of blood samples to be collected and the fraction of this to the total blood volume in the child were also used as previously reported [17].

The trial team met with the community again in the middle of the trial when preliminary results were presented on the data obtained from the mapping of the trial area such as number of houses, households, adults, children under one year and pregnant women. Other results shared at these meetings included laboratory data from screening of participants at enrollment such as community averages for haemoglobin, liver function tests, and electrolytes concentrations. Individual results were discussed privately with parents when requested. The trial team seized the opportunity of these meetings to address misconceptions regarding the use of blood samples and get feedback from the community. In addition, the final outcome of the trial was also shared with the community, at the end of the trial.

## Results

**Figure 2:**
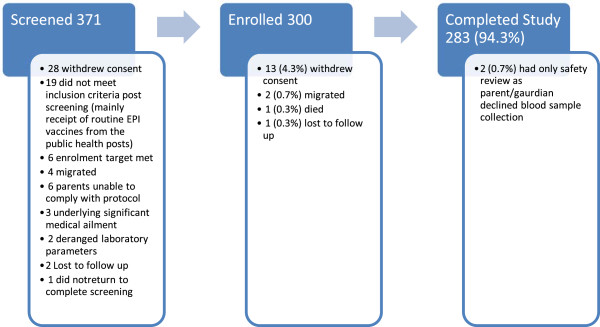
**Participant flow during the trial.**

**Figure 3:**
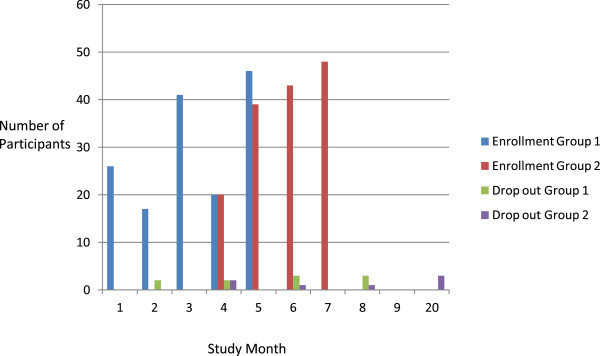
**Enrolment/Drop out rate.**

1044 potential participants received information regarding the trial of which 371 returned for screening.A total of 300 participants were recruited over a period of 8 months, with a follow up period of approximately 14 months per participant. 283 (94.3%) participants completed the trial (Figure 2). Of the 17 participants who did not complete the trial (Figure 3), 13 (75%) withdrew consent, 9 were due to family disputes over continued participation where a relevant member of the family had not been informed of the participants’ enrolment in the trial, and 4 were due to fear of continued blood sampling. 6 (47%) of those that withdrew consent were after 3 or fewer visits while the remaining 7 (53%) withdrawals were after 4 or more visits. Of the remaining 4 that did not complete the trial, 3 migrated out of the trial area and one died.

## Discussion

This trial recorded high retention rate of 94%, in a trial that had 6–9 scheduled visits, and 5–6 blood samples collected, within the 14 months duration of participation per enrolled participant. This was a remarkable success story, particularly for a new trial site, likely due to measures put in place by the trial team.

The handful of participants that could not complete the trial withdrew consent mainly due to an objection to further participation raised by a member of the family that was not present at the time of consent, or fear of subsequent blood sampling. This first reason brings to light a significant peculiarity within the region; the communal nature of relationships which may affect the consent process. The legal age in the Gambia is 18 years, and parents at this age can give consent for themselves or their children when applicable. It is clear however that in this context, other family relationships still play a significant role. Thus while one parent; usually the mother signs the consent form, it is wise to have trial staff engage other family members with a significant say in the life of the child such as fathers, grandfathers or grandmother prior to participant enrolment.

The use of mobile phones which has recently gained prominence in field trials with use ranging from contact, to education and data transmittal [18, 19] is likely to have contributed to the success of this trial. In an era where almost every home no matter how remote can boast of a mobile phone [20–22] it has become a resource which the public health community, including this trial, cannot ignore. Use in this trial was limited to contacting the participants, but it is likely to have significantly reduced overall contact costs and helped to ensure participant retention as it created an avenue to keep in close contact with participants. Mobile phones also served to help participants contact the trial staff whenever they had concerns. This may have enhanced the confidence of the participants as their concerns could be addressed immediately without waiting for the next clinic appointment which could have also impacted retention positively. This coupled with the numbering of houses and availability of basic demographic data made it easy to track participants, to address issues, perform home visits and maintain confidence in the trial team. In a society where living is so communal addressing concerns early was key to ensuring that confidence in the team was maintained within the community.

Comparable numbers withdrew during the early and late parts in the follow up phase suggesting that duration of trial participation did not play a major role in the decision to complete the trial. The use of a participant tracking database also helped to ensure that despite a complicated visit schedule varying between 6 sub-groups, participant visits were not missed. This was particularly key considering the low literacy levels [23] within the region where very few participants would return on the basis of an appointment alone. This also helped the trial team to plan workload in terms of resources needed weekly within the field. Such planning minimized participant waiting time which may have also contributed to the high retention rates. The prior mapping of the community and use of diagrams to identify homes ensured that despite the lack of an address system in the locality the trial team was able to trace participants, thus minimizing drop-outs due to inability to trace participants.

Screening out participants with parents/guardians perceived to be unable to comply with the protocol could also have played a role. This included ensuring that criteria such as presence within the trial area throughout the trial duration, including planned short term/long term travel, and previous utilization of immunization services were fulfilled.

Blood sampling remains a major challenge in conducting clinical trials in West Africa, with more resistance often encountered when larger blood volumes are collected [3, 24]. This factor also came to bear in this trial being the only other reason for consent withdrawal, with some subjects who completed the trial declining blood sampling while accepting safety review. Constant education including the use of illustrations is likely to have minimized this effect. Important to note however is the spread of information within communities often fuelled by communal living. At some point in this trial, the trial team became aware that rumors were circulating in the community that blood samples from trials were sold in Europe for monetary gain. Community engagement through community meetings, sharing preliminary demographic and clinical laboratory results with the community as well as taking representatives of the community to witness the laboratory processing of the blood samples helped to dispel such rumors. This was likely further strengthened by the involvement of community leaders in the community engagement sessions. The involvement of these leaders was crucial to gaining community acceptance of the trial which is crucial in these settings. Key and opinion leaders have been documented to play a significant role in acceptance of public health interventions [25, 26]. Following trial completion, feedback on results has also been given to the community. These meetings were useful for both the trial team and the community in particular as that was the first time these parameters were known to them. This will likely also have a positive impact on future trials.

## Conclusion

Participant retention in clinical trials remains a crucial factor in ensuring generalisabilty of trial data. This trial with retention of 94.3% in a trial in which a participant remained for 14 months, with rigorous scheduled visits and blood sampling is a huge success. This excellent retention can be ascribed mainly to continuous community involvement, adequate explanation of trial procedures, risks/benefits, and innovative tracing of participants adapted for the setting. It is hoped that these will guide others developing new trial sites or reviewing the operations at existing sites.
